# A High-Precision Automatic Pointer Meter Reading System in Low-Light Environment

**DOI:** 10.3390/s21144891

**Published:** 2021-07-18

**Authors:** Xuang Wu, Xiaobo Shi, Yongchao Jiang, Jun Gong

**Affiliations:** 1 College of Information Science and Engineering, Northeastern University, Shenyang 110819, China; 20182380@stu.neu.edu.cn (X.W.); 20182327@stu.neu.edu.cn (X.S.); 20181789@stu.neu.edu.cn (Y.J.); 2 Institute of Image Recognition and Machine Intelligence, Northeastern University, Shenyang 110819, China

**Keywords:** automatic meter recognition, skew correction, illumination enhancement fusion algorithm, needle direction regression

## Abstract

At present, pointer meters are still widely used because of their mechanical stability and electromagnetic immunity, and it is the main trend to use a computer vision-based automatic reading system to replace inefficient manual inspection. Many correction and recognition algorithms have been proposed for the problems of skew, distortion, and uneven illumination in the field-collected meter images. However, the current algorithms generally suffer from poor robustness, enormous training cost, inadequate compensation correction, and poor reading accuracy. This paper first designs a meter image skew-correction algorithm based on binary mask and improved Mask-RCNN for different types of pointer meters, which achieves high accuracy ellipse fitting and reduces the training cost by transfer learning. Furthermore, the low-light enhancement fusion algorithm based on improved Retinex and Fast Adaptive Bilateral Filtering (RBF) is proposed. Finally, the improved ResNet101 is proposed to extract needle features and perform directional regression to achieve fast and high-accuracy readings. The experimental results show that the proposed system in this paper has higher efficiency and better robustness in the image correction process in a complex environment and higher accuracy in the meter reading process.

## 1. Introduction

Pointer meters are widely used in electric power systems, petrochemicals, military industries, and other fields. The current main method of meter reading is manual meter reading, but it has the problems of low efficiency, poor accuracy, and inability to detect in real-time. Therefore, the current main trend is to use machine vision-based inspection robots instead. However, the existing algorithms cannot solve the problems of tilt, distortion, low light, and uneven light illumination. There is an urgent need to utilize the latest technology to improve recognition accuracy and facilitate the unattended process.

Most of the early methods were based on computer vision for a meter reading. Jian et al. [1,2] combined binary image subtraction and Hough transform to estimate the angle of the needle and used the angle method to calculate the meter value. Bao et al. [3] used an inverse perspective mapping method to align the images of the meter to improve the needle position detection. Liu et al. [4] used a region growing method and hit-or-miss method to improve the segmentation of the needle region. Ma [5] then proposed a random sample consistency method to overcome background image interference. These methods run faster, but the traditional target detection and segmentation algorithms are less accurate and less robust. With the rise of deep learning, its application has led to a significant increase in the accuracy of meter detection and reading recognition. Fang et al. [6] proposed detecting meters using Mask R-CNN [7] to detect needle and scale key points and calculate readings by semantic segmentation. Alexeev [8] proposed a NiN-based AMR detector to detect pointer meters with different analog ranges, automatically. Salomon et al. [9] detected the bounding box of the dial by YOLO-based methods, and Faster-RCNN extracted the needle and then calculated the readings. These methods can be effective, but the detection success rate and reading accuracy are significantly degraded under disturbances such as tilt and uneven illumination, and preprocessing is needed for improvement.

Most of the captured meter images suffer from skew due to shooting angle and meter mounting position. Zheng et al. [10] used a multiscale homomorphic filtering algorithm to reduce the effect of luminance on the image and a perspective transformation to correct the image for more robust needle recognition. Xu et al. [11] used geometric transformation to correct the scene image and obtained the meter sub-image for reading after segmentation. Zhou et al. [12] used YOLOv3 to extract the dial key points and then used the least-squares method to perform ellipse fitting, and then used the perspective transformation for correction. Zhang et al. [13] used Faster-RCNN to detect and classify the meter and correct the meter image based on the edge line slope, but this method is only applicable to meters with straight-edge dials. Li et al. [14] proposed an algorithm for instrument image correction based on text position, which applies to many types of instruments. The methods mentioned above have a high correction success rate. However, the premise is that the paved obtained images have good quality, i.e., the above correction methods cannot cope with the reading requirements in complex environments.

Instrumented images acquired by robots working in rainy, fog, and nighttime environments are often weakly and unevenly illuminated. Retinex theory allows for simultaneous compression of dynamic range, preservation of color constancy, and hue reproduction enhancement, which is particularly suitable for low-light image enhancement. In recent years, many improvements based on this theory have been proposed. These include Bolun Cai et al. [15], who proposed an a priori information constraint, and Fu et al. [16], who used adaptive histogram equalization to adjust contaminated images. Thepade et al. [17] proposed a robust Retinex model and a priori a weighted fusion enhancement scheme. Recently, Wei et al. [18] combined it with neural networks to design a Retinex network, including a De-com network for decomposition and an enhancement network for illumination adjustment. Huang et al. [19] introduced an improved attention mechanism. The addition of deep learning dramatically improves the enhancement effect, but they both require a large number of suitable micro-illuminated image datasets, which is impossible to achieve in most cases. Current image processing methods cannot fully extract instrumental information with only one low-light enhancement process, and some schemes suffer from distortions such as overexposure and visual artifacts.

In this paper, an automatic meter reading system in complex environments based on computer vision and deep learning is constructed. The system is designed for different types of circular pointer meters. A pointer meter image skew correction algorithm based on improved Mask-RCNN and binary masks is proposed. The Faster-RCNN is improved by adding centrality constraints based on the attention mechanism, which improves the accuracy of automatic meter recognition and reduces the training cost by transfer learning. Based on PrRoI Pooling improved binary mask extraction, high-precision ellipse fitting and tilt correction are achieved. Then, an RBF illumination enhancement fusion algorithm based on improved Retinex and Fast ABF is proposed to effectively eliminate low-light interference. Finally, we use the improved ResNet101 network to extract the needle and perform needle direction regression to achieve fast and highly accurate meter readings.

Our main contributions are as follows:

(1) A scheme for an automatic meter reading system based on computer vision and deep learning in complex environments is proposed.
(2) A pointer instrument image skew-correction algorithm based on binary mask and improved Mask-RCNN is proposed to achieve high-accuracy ellipse fitting and tilt correction.
(3) The RBF illumination enhancement fusion algorithm is proposed to effectively eliminate low-light interference.
(4) The improved ResNet101 is proposed to extract needle features and perform directional regression for fast and highly accurate readings.

The rest of the paper is organized as follows. Section 2, Section 3 and Section 4 describe in detail the tilt correction, low-light enhancement, and readout recognition modules of our automatic recognition system, respectively. Section 5 provides the experimental results corresponding to each section. Finally, Section 6 draws conclusions.

## 2. Improved Mask-RCNN-Based Skew-Correction of Pointer Instrument Image

We designed Mask-RCNN with two phases. The first stage uses a Region Proposal Network to generate candidate object boxes, and the second stage is composed of a Binary Mask Prediction Branching Network and a Faster-RCNN classifier. Finally, the skew correction is completed by the perspective transformation based on the binary mask.

### 2.1. Improved Faster-RCNN Based on Attention Mechanism

There is no publicly available, common, real dataset for substation scenarios in the field of pointer instrument testing. Most of the available datasets are simulated datasets collected in a laboratory environment. Due to the non-negligible differences, when we apply the instrumentation detection model Faster-RCNN trained on the simulated dataset to the real dataset, the performance of the model has a significant degradation of varying magnitude.

To avoid classification confusion due to incomplete simulation, we need to make more use of the dial information so that the model shifts its attention more from the instrument housing to the instrument dial.

In this paper, we add centrality constraints to the meter image features based on the principle of attention mechanism [20]. Specifically, the feature map of the meter is multiplied by a mask expressed as *H*, which takes the form of a Butterworth filter with a large response at the center of the meter and a small response at the periphery of the meter. The mathematical expression is shown in Equation (1). *A*, *B* are the height and width of the instrument feature map, (x, y) is the spatial coordinate on the feature map, and *α* is the normalized radius of the mask area.

(1) $$H=\frac{1}{1+\frac{{\left(\frac{x}{B}\right)}^{2}+{\left(\frac{y}{A}\right)}^{2}}{\alpha^{2}}}$$

Additionally, in order to reduce the amount of data required for the new task and reduce the training time, a transfer learning-based model training method is used in this paper. Specifically, we first initialized our network based on the COCO dataset [21], which is mainly for the object detection task. By freezing most of the previous convolutional layers and replacing the last layers of the network, the output dimension matches the new task, and the parameters are initialized.

**Figure 1:**
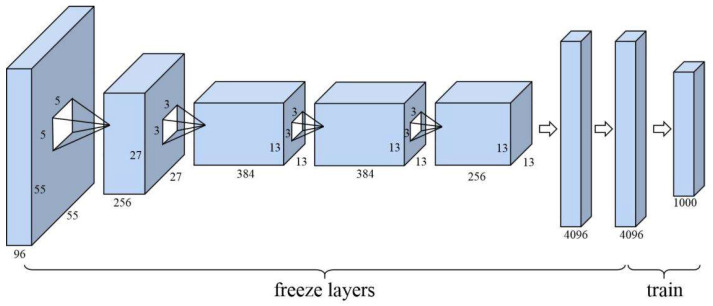
Transfer learning principles diagram.

As shown in Figure 1, training on a new task dataset with a smaller learning rate allows the model to obtain better performance than random initialization for large-scale data learning.

### 2.2. Binary Mask Extraction Based on PrRoI Pooling

**Figure 2:**
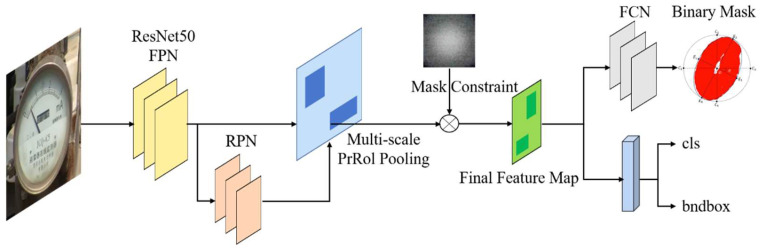
Flow chart of the first stage of mask extraction.

**Figure 3:**
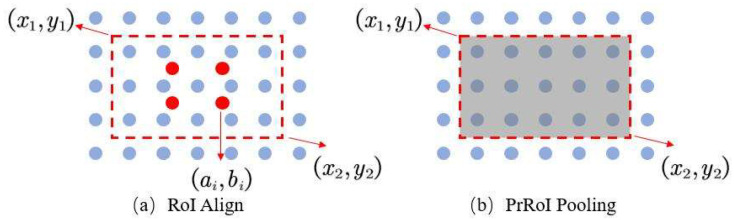
Comparison of pooling methods. (**a**) The RoI Align sampling a fixed number of points. (**b**) The PrRoI Pooling using a fully integrated averaging pool.

As shown in Figure 2, after generating the candidate object bounding boxes in the first stage, the feature map is bilinearly interpolated to make it continuous, then pooled with PrRoI Pooling [22] to obtain the size-fixed feature map, and finally centered and constrained to obtain the final feature map, which is then flattened and passed through the fully connected layers to output the meter classes and meter locations. The second stage lets it obtain the binary masks for instrument dials through the image segmentation network. Specifically, the final feature map obtained by PrROI Pooling and feature constraints is used as the input of the instance partitioning network FCN, and the output of the FCN network is the mask corresponding to the meter dial. The principle is shown in Figure 3.

As shown in Figure 3, in this paper, we use PrRoI Pooling instead of RoI Align [23] in traditional Mask-RCNN. PrRoI Pooling is an averaging pooling method based on bilinear interpolation to make the image contiguous. In contrast to RoI Align, it uses a fully integrated averaging pool instead of sampling a fixed number of points on it, thus making the coordinates of the final feature map continuous. Experiments show that using this measure can improve the accuracy of subsequent mask extraction.

We use F to denote the feature map, and $F_{i,j}$ denotes a discrete feature point in the feature map, then the feature value at the continuous coordinate (x,y) of the bilinear interpolation can be expressed as:

(2) $$F\left(x,y\right)=\underset{i,j}{\sum }C\left(x,y,i,j\right)\times F_{i,j}$$

(3) C(x,y,i,j)=max(0.1−|x−i|)×max(0.1−|y−j|)

$\left[\left(x_{1},y_{1}\right),\left(x_{2},y_{2}\right)\right]$ is used to denote a bin in the continuous *F*. Then, the average pooling result of a bin can be expressed as:

(4) $$Pr=\frac{\int_{y_{1}}^{y_{2}}\int_{x_{1}}^{x_{2}}F\left(x,y\right)dxdy}{\left(x_{2}-x_{1}\right)\times \left(y_{2}-y_{1}\right)}$$

After the above operation, we obtain the size-fixed feature map, and after the centralization constraint, we obtain the final feature map, after which we go through the FCN network and output the binary mask of the meter dial.

PrRoI Pooling is superior to RoI Align in that it can solve the problem of a fixed number of interpolated pixels in RoI Align and has a continuous gradient of pixel values within each bin. PrRoI Pooling does not involve quantized rounding operations, which makes the feature map more accurate and complete in information.

### 2.3. Perspective Transformation Skew Correction Based on Binary Mask

#### 2.3.1. Coordinate Measurement

**Figure 4:**
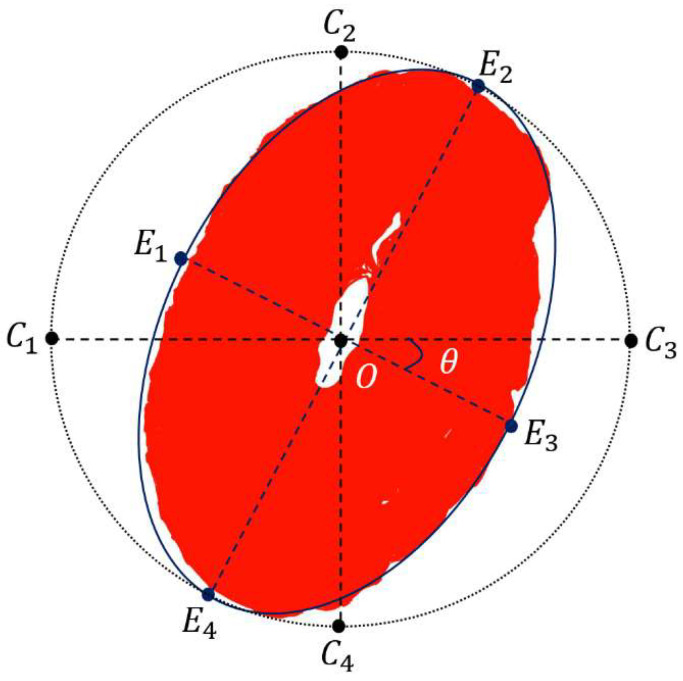
Calibration principle diagram.

Based on the dial binary mask extracted by Mask-RCNN, the ellipse fitting and perspective transformation correction are performed with better robustness than the ellipse fitting and perspective transformation correction based on traditional key point detection. The basic principle of calibration is shown in Figure 4. $C_{i}\left(i=1,2,3,4\right)$ denotes the intersections of the smallest external circle of the binary mask with the vertical and horizontal axes past the center of the circle, and $E_{i}\left(i=1,2,3,4\right)$ denotes the intersections of the minimum external ellipse of the dial binary mask with its long and short axes. $\left(C_{x},C_{y}\right)$ denotes the center coordinates of the external circle, *r* denotes the radius of the external circle, $\left(E_{x},E_{y}\right)$ denotes the center coordinates of the ellipse, (2a,2b) denotes the long and short axes of the ellipse, and *θ* denotes the deflection angle of the ellipse. The coordinates $\left(X_{i},\;Y_{i}\right)\;\left(i=1,2,3,4\right)$ of $E_{i}\left(i=1,2,3,4\right)$ are obtained from the following equations:

(5) $$X_{i}=E_{x}\pm \sqrt{E_{x}{}^{2}-\frac{Q}{1+k^{2}}},\;i=1,2,3,4$$

(6) $$Y_{i}=k\times X_{i}+q,\;i=1,2,3,4$$

(7) $$q=E_{y}-k\times E_{x}$$

(8) $$Q=E_{x}{}^{2}\times \left(1+k^{2}\right)-f$$

where Q, q are constants. When calculating the long axis intersections, k=tanθ, f=a, and when calculating the short axis intersections, $k=-\frac{1}{tan\theta },\;f=b.$ The coordinates of $C_{i}\left(i=1,2,3,4\right)$ can be calculated by $\left(C_{x}\pm r,\;C_{y}\pm r\right)$.

#### 2.3.2. Acquisition of Perspective Transformation Matrix

The process of calculating the perspective transformation is expressed as:

(9) $$\left[\begin{array}{c} X\\ Y\\ Z \end{array}\right]=T\times \left[\begin{array}{c} x\\ y\\ 1 \end{array}\right]$$

Mapping the three-dimensional coordinates to the two-dimensional space and expanding the matrix:

(10) $$x^{\prime }=\frac{X}{Z}=\frac{t_{11}\times x+t_{12}\times y+t_{13}}{t_{31}\times x+t_{32}\times y+t_{33}}$$

(11) $$y^{\prime }=\frac{Y}{Z}=\frac{t_{21}\times x+t_{22}\times y+t_{23}}{t_{31}\times x+t_{32}\times y+t_{33}}$$

where (x,y) denotes the coordinates of the ellipse intersection, $\left(x^{\prime },y^{\prime }\right)$ denotes the coordinates of the circle intersection, and the perspective transformation matrix is obtained by substituting the four pairs of points into the above equation. We use the matrix to correct the image tilt of the instrument dial.

## 3. Illumination Enhancement Based on RBF Fusion Algorithm

When the acquisition is performed on hazy, rainy days or at night, there are often low-light or uneven illumination problems in the meter images, which can significantly degrade the accuracy of subsequent meter readings. Therefore, this paper proposes an illumination enhancement fusion algorithm, which consists of an improved Retinex image enhancement algorithm and an improved adaptive fast bilateral filtering algorithm. The method can fully extract the dark content of the low-light region, preserve the meter texture details, and suppress the noise.

### 3.1. Improved Retinex Enhancement Based on Fractional Order and CRF

The conventional Retinex method suffers from over-enhancement and distortion when processing instrument images in different environments. In this paper, fractional-order differentiation is used instead of integer-order differentiation for improvement because it can effectively handle high-frequency components and texture detail information of low-frequency components. This can maximize the recovery of texture details and suppress noise in the low-light part of the instrument image.

In this paper, we use the Grunwald–Letnikov fractional order Dtλ Gf(t) [24], which is currently commonly used. After invoking the fractional-order differentiation for the reflection map *R*, the energy function is redefined as follows, where $\lambda_{1}$ and $\lambda_{2}$ are fractional-order parameters:

(12) E(R)=‖(Dxλ1 GR)λ2‖+‖(Dyλ1 GR)λ2‖

For the reflection constraint, according to Retinex theory, the illumination map *L* should contain the image structure information and not the texture details. Therefore, another illumination constraint is set as:

(13) E(L)=‖Dx GLφ+Gux(L)Dx G‖+‖Dy GLφ+Guy(L)Dy G‖

where G(L) is Gaussian filtered, and φ is a tiny positive number that prevents the denominator from being zero. Combining the prior illumination, the reflection constraint, and the fidelity term, the total energy function of the image is rewritten as:

(14) $$E_{s}\left(L,R\right)=\alpha E\left(L\right)+\beta {\left\|L-\tilde{L}\right\|}^{2}+\gamma E\left(R\right)+{\left\|LR-S\right\|}^{2}$$

where (α, β, γ) is the balanced equation weight coefficient. ${\left\|LR-S\right\|}^{2}$ is the fidelity used to ensure that image (L,R) is very close to the original image *S*. ${\left\|L-\tilde{L}\right\|}^{2}$ is the optical prior, where $\tilde{L}$ is the initial illumination map. Solving for $\underset{L,R}{\min}E_{S}\left(L,R\right)$, L and R are obtained to match the reflectance map constraints and optical prior information.

In order to meet the lightweight requirement of instrument identification, this paper chose Block Coordinate Descent (BCD) [25] based on the improved coordinate descent method to solve $\underset{L,R}{\min}E_{S}\left(L,R\right)$, which has significantly lower iteration cost, lower memory requirement, and easier parallelization than the commonly used gradient descent method.

Finally, to obtain the instrument target image with ideal exposure, the light intensity of the light exposure map needs to be adjusted. In this paper, Camera Response Function (CRF) transformation was used, where CRF adjusts each pixel to the desired exposure level based on the estimated exposure ratio map. The transformation equation is defined as:

(15) $$B\left(\tau ,p\right)=\mu L^{p}=p^{\tau^{a}}e^{b\left(1-\tau^{a}\right)}$$

where p is the input image, that is, the irradiation image L, τ is the exposure ratio, and (a,b) is the parameter set for the experiment according to the response function database [26] for images with different light only.

The CRF transform is closer to the real camera exposure, and the light image extracted by CRF should be closer to the real brightness of the original instrument image. Moreover, this improvement solves the problem of color and luminance distortion when the traditional Γ transform increases the visibility.

### 3.2. Improved Fast Adaptive Bilateral Filtering Based on Histogram Approximation

The illumination enhancement algorithm amplifies the ambient noise in the low-light region, and the convolution calculation leads to contrast reduction at the strong edges, which affects the subsequent feature extraction. Therefore, we use a bilateral filtering algorithm [27] that can both smooth the noise and better maintain the image edges and detailed texture regions.

We improve on the fast adaptive bilateral filtering algorithm by using a histogram approximation-based approach to speed up the computational process. The improved algorithm balances both speed and adaptivity, and achieves a speedup of at least 25 times, with no significant distortion in the image.

In the original method, since the grayscale distribution in the texture region near the image edges is not uniform, the local histogram is chosen to be approximated by matching the moments of the polynomial function and the local histogram. Define the histogram as follows:

(16) $$H_{i}\left(s\right)=\underset{j\in \Omega }{\sum }\omega \left(j\right)\delta \left[f\left(j\right)-\xi \left(i\right)\right],\;s\in \Theta_{i}=\left\{f\left(j\right):j\in \Omega \right\}$$

where (i,j) is the pixel centroid, ω(j) is the null domain kernel function, δ(s) is 1 at s=0 and 0 otherwise, and f(j) and ξ(i) denote the pixel value at the centroid of the region. The procedure approximates $H_{i}\left(s\right)$ by a polynomial and replaces the summation operation with an integral, transforming the solution of the original text as:

(17) $$M_{k}=\int_{0}^{1}{\left(s-\xi \left(i\right)\right)}^{k}\Phi \left(s-\xi \left(i\right)\right)ds,\;k=0,1,\ldots ,N+1$$

The final filtered image G(i) can be derived:

(18) $$\{\begin{array}{l} G\left(i\right)=f\left(i\right),\;\lambda_{min\;i}=\lambda_{max\;i}\\ G\left(i\right)=\lambda_{min\;i}+(\lambda_{max\;i}-\lambda_{min\;i})\cdot (\overset{N}{\underset{i=0}{\sum }}a_{i}M_{i})/(\overset{N}{\underset{i=0}{\sum }}a_{i}M_{i+1}),\lambda_{min\;i}\neq \lambda_{max\;i} \end{array}$$

where $\lambda_{min\;i}$ and $\lambda_{max\;i}$ are the upper and lower bounds of $\Theta_{i}$, and $a_{i}$ is the coefficient of the above matching polynomial. The integration replaces the summation operation while avoiding the inverse operation for the purpose of accelerating the above ABF.

### 3.3. RBF Illumination Enhancement Fusion Algorithm for Instrument Images

**Figure 5:**
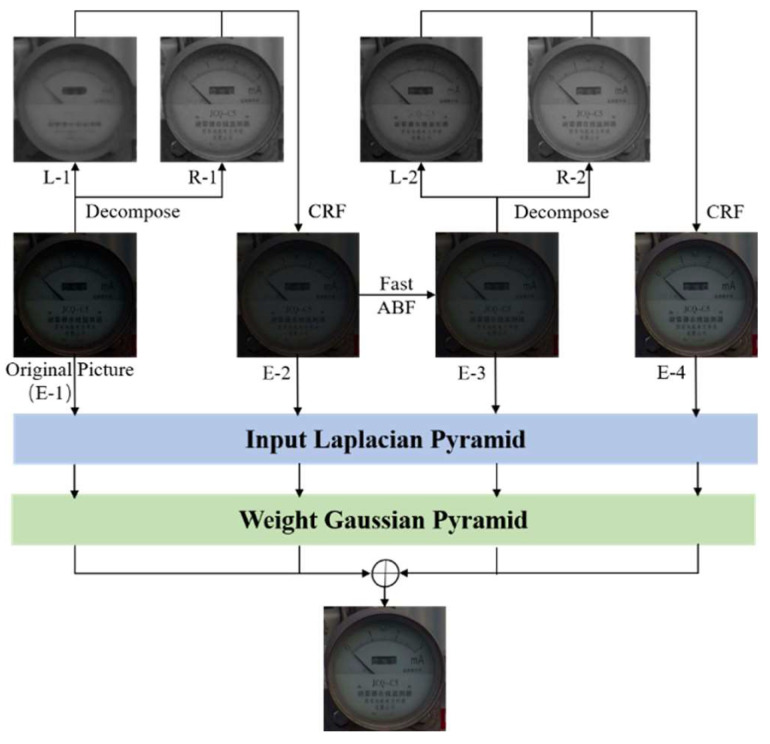
Schematic diagram of image enhancement fusion algorithm.

To maximize dark content enhancement and ensure contrast and noise suppression, we combined the above two algorithms to design the RBF low-light image enhancement fusion algorithm, where the algorithm process is as shown in Figure 5.

Firstly, the original low-light meter image E-1 is processed with the improved light enhancement algorithm, and the exposure is adjusted with CRF to obtain the first enhanced image E-2. Secondly, E-2 is filtered with the improved Fast ABF algorithm to obtain the enhanced image E-3 with clearer edge and texture details in the low-light area. In order to extract more dark content in the low-light region of the instrument image, a second low-light enhancement is applied to E-3, and an enhanced image E-4 with better exposure is obtained.

Finally, in order to achieve a good balance between enhancing brightness and avoiding overexposure, and to retain more details and textures in the original image, this paper performs Laplace pyramid decomposition and multiplies the corresponding Gaussian weight matrix for the original image E-1, enhanced images E-2, E-3, and E-4 respectively, and fuses them to obtain the final enhanced image with the following fusion equation:

(19) $$\Lambda \left(x,y\right)=\overset{4}{\underset{i=1}{\sum }}g\left(E_{i}\left(x,y\right)\right)\cdot m_{i}\left(x,y\right)$$

The original image E-1 contains all the details of the low-light meter image enhancement image and retains the highlighted areas. In contrast, E-4 has a certain degree of detail lost due to the filtering process but can show more dark content in the low-light areas than the original image E-1 and image E-2. In addition, the enhanced image E-3 not only reduces the noise interference compared to E-2 but also enhances the contrast using the neighborhood information.

Therefore, this paper uses the Sigmoid function to set the weights of E-1 and E-4. Since the pixel distribution of a well-exposed image is approximated as a Gaussian distribution with a mean of 0.5 and a variance of 0.25, the weights of E-2 and E-3 are set as Gaussian functions. To balance the Gaussian distribution function and the Sigmoid function, some changes are made to the standard formula to obtain the following weight formulas, as shown in Table 1.

Where $L_{i}$ is the corresponding light image, normalized by the weights calculated from the light image, and the RBF fusion algorithm significantly speeds up the filtering speed, enhances the contrast, and suppresses noise for subsequent needle feature extraction, while achieving maximum extraction of details in the low-light region of the instrument image.

## 4. Instrument Reading Based on Direction Regression

**Figure 6:**
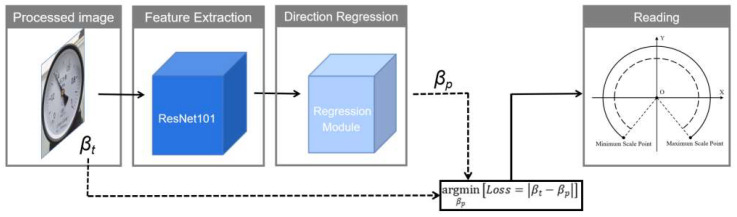
Flow chart of meter reading based on direction regression.

In the first two steps, we detect the meters in the image and correct them. The last step performs reading recognition. Traditional methods identify readings by detecting the needle and key points and then using the angle method, but the accuracy of the readings is overly dependent on the accuracy of key points’ positioning. This section introduces a method that directly regresses the needle orientation end-to-end using a deep neural network. Specifically, we designed a convolutional neural network-based needle direction regression model, including three steps: (1) extracting the needle by improved ResNet101, (2) regressing the needle direction by the direction regression module, and (3) obtaining the readings based on the needle prediction direction vector and angle correspondence. The flow chart of the method is shown in Figure 6.

Compared with the currently existing methods for pointer meter reading recognition, the method presented in this section does not explicitly detect the needle and key points, but takes advantage of deep learning in image processing to directly regress the direction of the needle end-to-end. This approach bypasses the tediousness and lack of accuracy of traditional methods in detecting needles and key points.

### 4.1. Instrument Needle Feature Extraction

Our goal is to regress the direction of the needle end-to-end. In this section, we propose the modified ResNet101 for needle feature extraction. Specifically, we use the part before the global averaging pooling layer of ResNet101 for feature extraction. The effect of feature extraction is essential for the accuracy of subsequent needle orientation regression. Since the global averaging pooling layer to find the global average operation is invariant to rotation, the directional information of the instrument space features is lost. Therefore, we make the following improvements to ResNet101: remove the global average pooling layer of ResNet101 and add a max pooling layer at the end of the network to compress the image feature size. This operation is to compensate for the global average pooling layer while efficiently extracting features.

### 4.2. Needle Direction Regression Module

After obtaining the needle features, based on the inspiration of the TextSnake regression text direction principle, this section processes the needle features by a direction regression module to obtain the direction vector of needle regression. The needle direction regression module consists of a 3 × 3 convolutional layer, a ReLU layer, a max pooling layer, and a fully connected layer. The convolution layer compresses the number of feature channels while processing the extracted features, while the max pooling layer reduces the spatial dimension of the features and the number of parameters in the fully connected layer, thus reducing the computation and improving the execution efficiency. Then, transition is performed through a flat layer to a fully connected layer, and a two-dimensional vector is output through the fully connected layer. Finally, the vector is normalized to obtain a unit vector for needle direction regression, denoted as v.

### 4.3. Instrument Reading Calculation

After obtaining the regression vector of the needle direction, we convert the vector to the angle in the plane right-angle coordinate system, and the conversion relationship is as in Equation (20), where $v_{x}$, $v_{y}$ are the horizontal and vertical coordinates of v, and the angle between the needle and the horizontal direction is noted as $\beta_{p}$. Then, according to the angle corresponding to the scale range of the type of meter used, the reading of the meter is calculated by Equation (21), where k is the predicted value of the meter’s reading, $\beta_{max}$ is the angle corresponding to the maximum scale of the meter, $\beta_{min}$ is the angle corresponding to the minimum scale, $k_{max}$ is the maximum scale value of the meter, and $k_{min}$ is the minimum scale. These parameters can be obtained in advance according to the specific type of meter.

(20) $$\beta_{p}={tan}^{-1}\frac{v_{y}}{v_{x}}$$

(21) $$k=\frac{\beta_{p}-\beta_{min}}{\beta_{max}-\beta_{min}}\left(k_{max}-k_{min}\right)+k_{min}$$

## 5. Results

### 5.1. Experimental Settings

#### 5.1.1. Dataset

- Simulation dataset: Currently, there is no common, large, real dataset in the field of meter detection. We first pre-trained the model based on the COCO dataset [21], after which 1024 images of scenes with meters were collected in a simulation environment for transfer learning. These 1024 images contain good images, underexposed, overexposed, Gaussian noise, pretzel noise, five categories of scenes, and the number distribution is {200,206,206,206,206}, and we also collected 256 images as a test set, for which the five categories of the number distribution are {40,54,54,54,54}.
- Real-world dataset: To test the recognition performance of the model in the field monitoring environment, we collected a total of 1672 images of various meters under Shijiazhuang substation with different tilt angles, different light interference, and random image quality. These data were used for model performance testing of target detection, mask extraction, angle regression, and light compensation algorithm comparison experiments.

#### 5.1.2. Experimental Environment

The experiments in this paper were run on a hardware environment with NVIDIA GTX3090 GPU and processor Intel(R) Core (TM) i7 CPU 2.3~4.6 GHz RAM 32 G. The algorithms in this paper were run under Win10 OS, Pytorch 1.7.1, Python 3.9, and cuda 11.0.

#### 5.1.3. Evaluation Indicators

- For an object detector, the most commonly used evaluation metric is mAP (mean Average Precision). To calculate the mAP, we need to set the IOU (Intersection Over Union) thresholds, which represent the ratio of the intersection and the union of the target prediction bounding box and the true bounding box. Therefore, this experiment uses mAP under different IOU threshold settings as the evaluation metric, and the three evaluation metrics are mAP (mean Average Precision under IOU taking 0.5:0.05:0.95), mAP^50^ (IOU = 0.5), and mAP^75^ (IOU = 0.75). For each category, we can find the corresponding AP (AP, AP50, AP75) values, and the mAP values are obtained after taking the arithmetic average over all categories. Specifically, for a category, the Precision and Recall under a given IOU threshold are calculated as shown in Equations (22) and (23), from which the P-R (precision-recall) curve is plotted, and the area under the curve is the AP value. According to this rule, we can calculate the AP value of each category, and then take the arithmetic average of all categories to obtain the mAP value under the three thresholds.
  (22) $$Precision=\frac{TP}{TP+FP}$$
  (23) $$Recall=\frac{TP}{TP+FN}\;$$
  where TP denotes the number of actual positive samples detected as positive, FP denotes the number of actual negative samples monitored as positive, and FN denotes the number of actual negative samples monitored as negative.
- For illumination enhancement algorithms, three image quality evaluation metrics were introduced to evaluate the natural image retention: Lightness Order Error (LOE) [28], Auto-Regressive-based Image Sharpness Metric (ARISM) [29] to evaluate the image sharpness, and Feature Similarity (FSIM) [30] to objectively assess the enhancement effect of different algorithms.

The relative order of the luminance can indicate the direction of the light source and the luminance change. The naturalness of the enhanced image is related to the relative order of the luminance in different local areas, and *LOE* is defined as follows:

(24) $$LOE=\frac{1}{N}\overset{N}{\underset{x=1}{\sum }}\overset{N}{\underset{y=1}{\sum }}\Psi \left(L\left(x\right),L\left(y\right)\right)⊕\Psi \left(\tilde{L}\left(x\right),\tilde{L}\left(y\right)\right)$$

(25) $$\Psi \left(x,y\right)=\{\begin{array}{l} 1\;,\;x\geq y\\ 0\;,\;Others \end{array}$$

where N is the total number of pixels, and L and $\tilde{L}$ are the maximum values of the color channels of the original and enhanced images, respectively. The value of LOE measures the naturalness of the retention between the original image and the enhanced image, and the smaller the value of LOE, the more natural the enhancement effect.

FSIM combines gradient similarity and color similarity to assess the enhancement effect, with higher values indicating greater similarity to a well-exposed image, as defined by:

(26) $$FSIM=\frac{\sum_{x\in \Omega }S_{L}\cdot PC_{m}\left(x\right)}{\sum_{x\in \Omega }PC_{m}\left(x\right)}$$

where $PC_{m}\left(x\right)$ is considered as a dimensionless measure of importance for the local structure and can be used as a weight measure factor.

ARISM can calculate the energy difference and contrast difference of Auto-Regressive (AR) model coefficients on each pixel separately, and then calculate the image clarity by percentile pooling to derive an overall quality score. Since ARISM considers both image luminance and color information in the auto-regressive parameter space to evaluate the effect of enhancement, a lower value indicates a clearer image.

### 5.2. Experimental Results

#### 5.2.1. Instrument Detection and Calibration Experiments

- Model training based on transfer learning

The performance comparison results of Mask-RCNN networks obtained by two training methods with and without transfer learning are shown in Table 2, in which the feature extraction networks were used with ResNet50-FPN and ResNet101-FPN respectively, and the target detection and instance segmentation of the models trained in the four cases were performed on real datasets under the same conditions as other experiments. The performance of the models trained in the four cases was tested on real datasets under other experimental conditions. The table shows that the performance of the models trained with transfer learning was improved for both target detection and mask extraction, and the feature extraction network achieved better performance with ResNet101-FPN than with ResNet50-FPN. The target detection AP50 is 2.7 percentage points higher for the network based on pre-trained ResNet101-FPN than for the network based on no pre-trained ResNet50-FPN, and the mask extraction AP50 is 4.1 percentage points higher.

- Comparison of network performance after adding PrRoI Pooling

The four Mask-RCNN networks in Table 3 were trained using transfer learning under the same other conditions, and the target detection and instance segmentation performance of the networks were tested on real datasets. As shown in Table 3, the results indicate that using PrRoI Pooling instead of RoI Align to obtain the feature map can improve the performance of Mask-RCNN, and the performance was improved. The performance improvement was more obvious when the ResNet101-FPN feature extraction network was used, for example, the target detection AP50 improved by 3.1% when the ResNet101-FPN was used, while the target detection AP50 decreased by 0.5% when the ResNet50-FPN was used instead.

- Comparison of network performance after adding feature constraints

On top of replacing RoI Align in Mask-RCNN with PrRoI Pooling, feature constraints were added to the network, and the four Mask-RCNN networks in Table 4 were trained using transfer learning, with other conditions being the same. The performance of the networks was tested on real datasets. As shown in Table 4, the results indicate that the models’ target detection and mask extraction performance were significantly improved after the inclusion of feature constraints.

- Instrument dial tilt-correction results based on binary mask

Based on the above comparative experimental results, it is clear that our improved method is feasible and effective.

(1) Training the network using transfer learning will improve the performance of the model, and using the ResNet101-FPN feature extraction network can lead to better performance of the model than using ResNet50-FPN. Meanwhile, by replacing RoI Align with PrRoI Pooling, the performance is further improved, and the ResNet101-FPN has a more obvious improvement effect than ResNet50-FPN. Combining the above two reasons, we decided to use ResNet101-FPN for the subsequent experiments.
(2) Further addition of feature constraint sessions for Mask-RCNN revealed further significant improvement in model performance. Finally, to compare the availability of our overall improvement scheme, the engineering metric availability was introduced, which is defined as follows:
  (27) $$a=\frac{count\left(3\%\right)}{count}$$
  where count denotes the number of recognized corrected meter images, and count(3%) denotes the number of recognized images where the relative error of the readings is less than 3%. The method of reading here is as described in Section 4. A comparison of the availability of our improved scheme with the conventional scheme is shown in the following Table 5.

**Figure 7:**
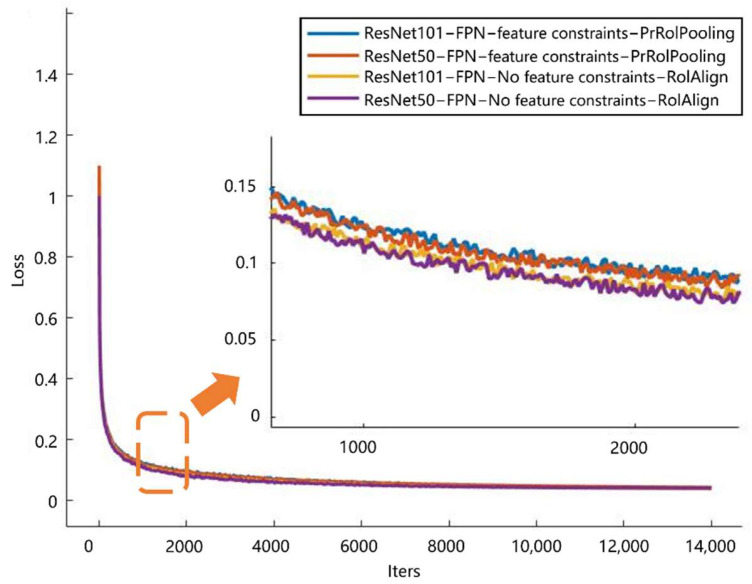
Iterative loss curves for different models.

Meanwhile, we obtained the iterative loss curves for the four models in the table, as shown in Figure 7. The curves show that our improved scheme led to a significant improvement in model performance, with essentially no increase in training time.

To synthesize the above conclusions, we used ResNet101-FPN in the improved Mask-RCNN to extract image features, replaced RoI Align with PrRoI Pooling, added feature constraints, and trained the model using the transfer learning method. We applied the trained model to the real-world dataset to obtain the binary mask of the instrument dial, which in turn yielded the perspective transformation matrix, and applied this matrix again to the instrument dial to correct the tilt of the dial.

**Figure 8:**
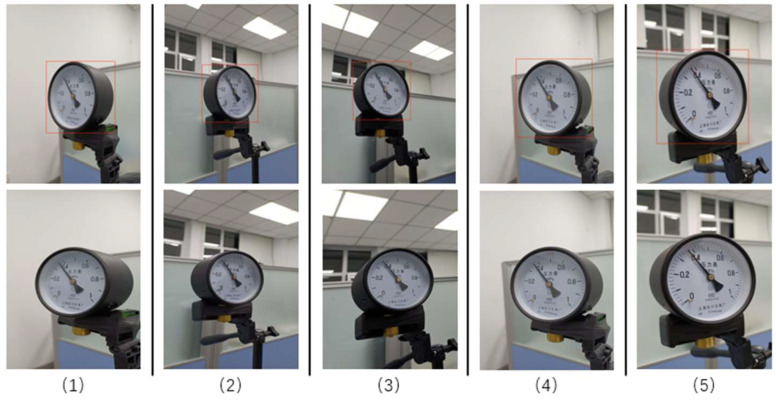
Skew correction results.

As shown in Figure 8, five pointer meter images with different tilt angles were selected and calibrated to demonstrate the feasibility and effectiveness of the perspective transformation method based on binary masks.

#### 5.2.2. RBF Illumination Enhancement Experiment

After successfully detecting the gauge dials and calibrating them, it is necessary to take readings, but the low-light environment can lead to a significant decrease in reading accuracy. In this paper, we showed the process and effect of the improved light enhancement fusion algorithm for pressure gauge image enhancement in Figure 5. To further verify the effectiveness of the improved algorithm proposed in this paper, we compare the results with Contrast Limited Adaptive Histogram Equalization (CLAHE) [31], Dark Channel Defogging (HRDCP) [32], Multi-Scale Retinex with Color Restoration (MSRCR) [33], and MSRCR + gamma [34] in the same experimental environment.

**Figure 9:**
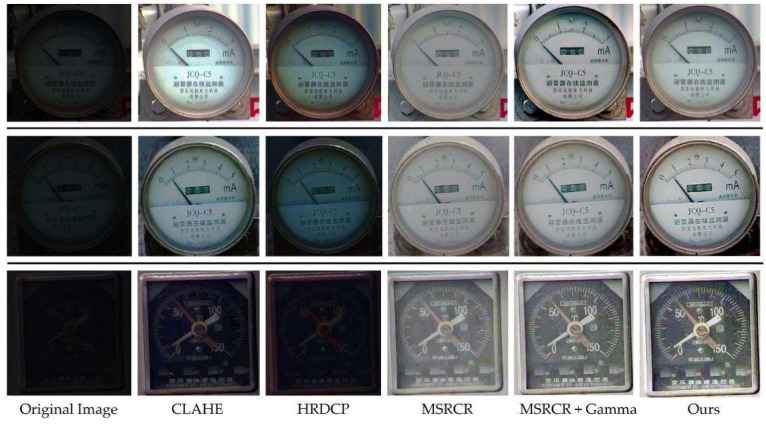
Comparison of the effect of each light enhancement algorithm.

The results of light enhancement experiments for each instrument image under different low-light conditions are shown in Figure 9.

Affected by the night environment, the global brightness of the original image is low, and the dial scale is not clear. The enhancement result by CLAHE brings artifacts and loses some details. The overall exposure effect of the enhancement by dark channel defogging was dark. For the traditional MSRCR-based enhancement results, it can be found that the enhancement effect was better after the gamma transformation, but there was overexposure in all of them. In this paper, the low-light image enhancement algorithm greatly reduced the color and luminance distortion, making the details of the meter part clearer, while preserving the color details of the target image. That is, we achieved a good balance among brightness, exposure, and contrast. While avoiding overexposure, we greatly improved the brightness and contrast of key parts of the meter images.

In order to compare the advantages and disadvantages of our algorithm with other algorithms more objectively and visually, the evaluation metrics of each algorithm were calculated, as shown in Table 6.

As can be seen from the table, the RBF light enhancement fusion algorithm proposed in this paper achieved better results in all three evaluation metrics. It outperformed other comparison algorithms in all aspects of image naturalness preservation, image sharpness, gradient similarity, and color similarity. It should be noted here that the overall enhancement effect of the dark channel defogging algorithm was poor and very close to the original low-light image, resulting in a very low LOE value, which cannot indicate that the dark channel defogging algorithm has a more natural enhancement effect.

#### 5.2.3. Deep Learning-Based Needle Angle Regression Experiment

- Instrument needle direction regression

We obtained the meter reading using the needle direction regression module to get the angle between the needle and the horizontal direction. To fully illustrate the superiority of the end-to-end regression needle orientation method using neural networks, we performed a comparison experiment with the traditional methods.

**Figure 10:**
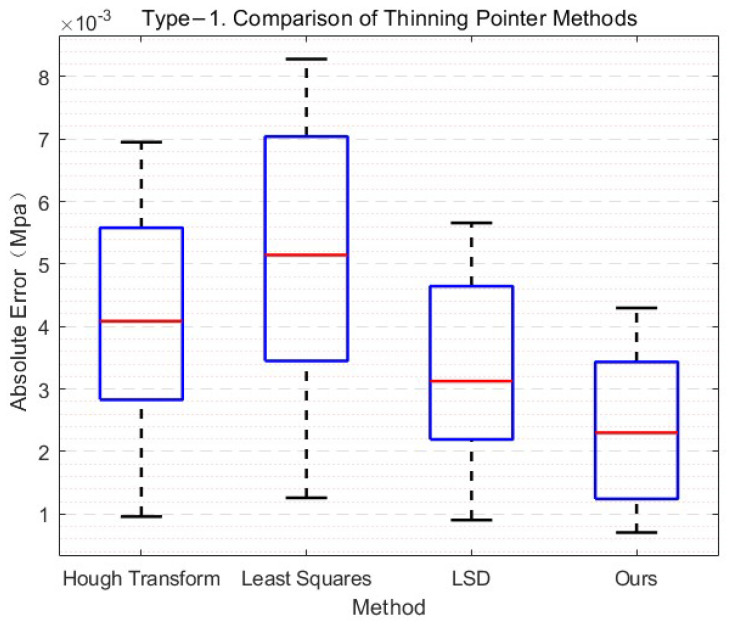
Refined needle method performance comparison.

For this purpose, we constructed a test set containing 300 samples with standard indices of 0.2, 0.4, 0.6, 0.8, and 1.0. We performed comparison experiments under the same conditions several times. The experimental results showed that the error of our algorithm was lower than that of the other three compared algorithms. To intuitively reflect the performance of the algorithm, we visualized the experimental data, as shown in Figure 10. By analyzing the data, we can see that the overall performance of our algorithm was better than other algorithms and can meet the requirements of practical engineering applications.

**Figure 11:**
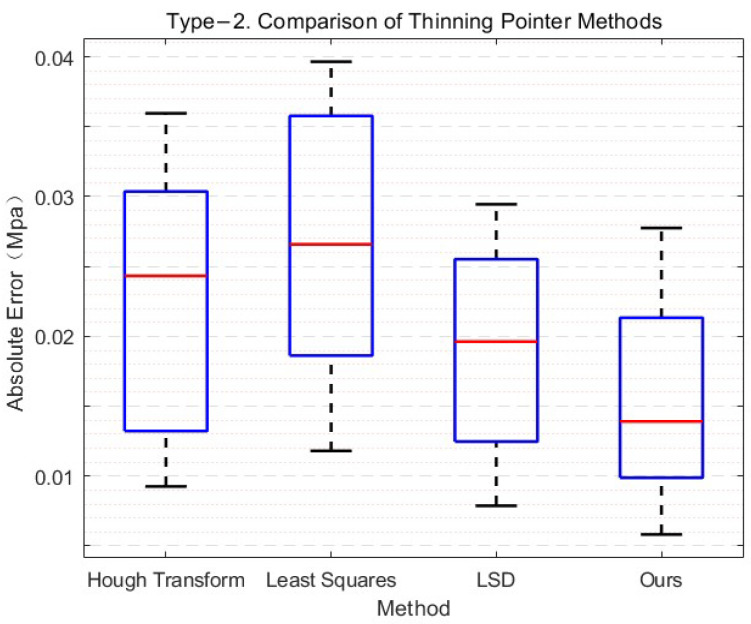
Robustness testing of refined needle methods.

In addition to this, we also tested the robustness of the algorithm. Specifically, we conducted experiments on different types of circular meters and recorded the experimental data visualization in Figure 11. The result shows that the algorithm is robust to different types of circular meters.

- Comparative experiment of automatic meter reading recognition scheme

Finally, to illustrate the superiority of the automatic meter reading scheme proposed in this paper, we performed a comparison test with the classical schemes in this field. We selected methods from Chi et al. [35], Kucheruk et al. [36], and Gao et al. [37]. Since there is no unified evaluation metric in this field, some methods usually use the mean reading error for algorithm performance measurement. To reflect the overall performance of the algorithm, we used the mean relative error, $\bar{\delta }$, and the mean reference error, $\bar{\gamma }$, as evaluation metrics. $\bar{\delta }$ and $\bar{\gamma }$ are defined as follows:

(28) $$\bar{\delta }=\frac{1}{N}\overset{N}{\underset{i=1}{\sum }}\frac{\left|k-k_{t}\right|}{k_{t}}\times 100\%$$

(29) $$\bar{\gamma }=\frac{1}{N}\overset{N}{\underset{i=1}{\sum }}\frac{\left|k-k_{t}\right|}{k_{max}-k_{min}}\times 100\%$$

where $k_{t}$ is the actual value, *k* is the reading value, and N is the total number of samples in the dataset. The experimental results are shown in Table 7.

The experimental results show that the mean relative error of our algorithm is 2.217%, which is slightly smaller than the 2.473% of Chi et al., but the mean reference error is much better than the other three methods. In addition, our method avoids the process of explicitly detecting the key points of the instrument by traditional algorithms, but combines the overall information of the instrument image from the data to improve the anti-interference capability and weaken the reading error caused by inaccurate detection and positioning of the key points, thus enabling a more accurate return of the instrument readings.

## 6. Conclusions

This paper proposed an automatic reading system based on computer vision and deep learning for different types of pointer meters in the low-light environment. By integrating the proposed tilt-correction and RBF illumination enhancement algorithms into the application, the images acquired under interference can be quickly compensated and corrected to standardized recognition images. Further, we used ResNet101 to extract needle features and performed needle direction regression to accurately calculate meter readings.

In the tilt-correction process, the Faster-RCNN in the improved Mask-RCNN model was used to detect the meter first. After adding PrRoI Pooling and centering constraints, the binary mask of the meter dial was obtained by FCN. Then, high-precision ellipse fitting was performed to obtain the perspective transformation matrix and complete the tilt correction. For the low-light compensation part, we first improved Retinex based on fractional order and CRF, and then combined it with the improved Fast ABF to form the RBF light enhancement fusion algorithm. Finally, the needle features were extracted by improving ResNet101, end-to-end regression was performed for the needle direction, and the display number was read based on the angle method.

The experimental results show that the system achieved high speed, high accuracy, and a high success rate in the process of tilt correction and light compensation. At the same time, the system can automatically read different types of pointer instruments with much higher reading accuracy than manual inspection. More importantly, the robustness and recognition accuracy of our system for different complex environments were also confirmed to meet the requirements of unattended applications.

It should be specified that our system has a different degree of degradation of recognition for meter images in rainy and foggy weather conditions. This is mainly due to the limited ability of the Retinex algorithm to eliminate rain and fog interference. For recognizing the interference of foreign object occlusion in the field, no corresponding solution has been proposed in this paper. In addition, our system had good recognition for most circular single-pointer meters, but limited recognition for multi-pointer meters. This is also our next breakthrough target.

## Figures and Tables

**Table 1:** Weight formulas for RBF light enhancement fusion algorithm.

*m*_1_	*m*_2_	*m*_3_	*m*_4_
$\frac{1}{e^{-4L_{1}+2}+1}$	$e-\frac{{\left(L_{2}-0.5\right)}^{2}}{2\cdot 0.25^{2}}$	$e-\frac{{\left(L_{3}-0.5\right)}^{2}}{2\cdot 0.25^{2}}$	$\frac{1}{e^{4L_{4}-2}+1}$

**Table 2:** Model performance obtained with different training methods.

Model	Training Time (h)	Target Detection			Mask Extraction		
		mAP	mAP^50^	mAP^75^	mAP	mAP^50^	mAP^75^
No pre-training ResNet50-FPN	1.35	0.726	0.834	0.803	0.631	0.811	0.637
Pre-training ResNet50-FPN	1.13	0.734	0.872	0.812	0.647	0.863	0.652
No pre-training ResNet101-FPN	1.77	0.747	0.832	0.813	0.665	0.823	0.658
Pre-training ResNet101-FPN	1.45	0.763	0.861	0.839	0.672	0.852	0.674

**Table 3:** Model performance obtained with different pooling methods.

Model	Target Detection			Mask Extraction		
	mAP	mAP^50^	mAP^75^	mAP	mAP^50^	mAP^75^
ResNet50-FPN RoI Align	0.734	0.872	0.812	0.647	0.863	0.652
ResNet50-FPN PrRoI Pooling	0.743	0.867	0.830	0.656	0.869	0.669
ResNet101-FPN RoI Align	0.763	0.861	0.839	0.672	0.852	0.674
ResNet101-FPN PrRoI Pooling	0.773	0.892	0.851	0.683	0.872	0.685

**Table 4:** Model performance obtained with or without feature constraints.

Model	Target Detection			Mask Extraction		
	mAP	mAP^50^	mAP^75^	mAP	mAP^50^	mAP^75^
ResNet50-FPN No feature constraints	0.743	0.867	0.830	0.656	0.869	0.669
ResNet50-FPN feature constraints	0.768	0.906	0.864	0.685	0.902	0.680
ResNet101-FPN No feature constraints	0.773	0.892	0.851	0.683	0.872	0.685
ResNet101-FPN feature constraints	0.826	0.938	0.868	0.712	0.909	0.727

**Table 5:** Availability of different models.

Model	Threshold	Availability
ResNet50-FPN–No feature constraints–RoI Align	3%	0.961
ResNet50-FPN–feature constraints–PrRoI Pooling	3%	0.973
ResNet101-FPN–No feature constraints–RoI Align	3%	0.969
ResNet101-FPN–feature constraints–PrRoI Pooling	3%	0.988

**Table 6:** Results of light enhancement metrics for each algorithm.

Method	LOE			FISM			ARISM		
CLAHE	975	1081	1752	3.741	3.522	3.988	0.805	0.747	0.736
HZDCP	98	119	84	3.903	3.534	4.141	0.807	0.644	0.705
MSRCR	917	583	975	3.628	3.354	3.482	0.831	0.768	0.821
MSRCR + Gamma	564	309	867	3.535	3.246	3.368	0.851	0.798	0.843
RBF	428	235	304	3.284	2.836	3.054	0.943	0.912	0.884

**Table 7:** Comparison of the accuracy of pointer meters’ reading recognition.

Method	$\bar{\delta }$ (%)	$\bar{\gamma }$ (%)
I [35]	2.473	0.613
II [36]	3.56	1.372
III [37]	2.912	0.573
Ours	2.217	0.208

## Data Availability

Not applicable.
